# SMART HOME DATA VISUALIZATION FOR PROACTIVE HEALTH MONITORING OF COMMUNITY DWELLING OLDER ADULTS

**DOI:** 10.1093/geroni/igac059.2233

**Published:** 2022-12-20

**Authors:** Katherine Wuestney, Javier Ramirez, Diane Cook, Roschelle Fritz

**Affiliations:** Washington State University, Vancouver, Washington, United States; Washington State University, Pullman, Washington, United States; Washington State University, Pullman, Washington, United States; Washington State University, Vancouver, Washington, United States

## Abstract

The role of Ambient Assistive Living and smart home technologies, which utilize unobtrusive sensors to detect changes in health, is becoming increasingly important in the delivery of healthcare services to older adults. However, these technologies must be designed to meaningfully incorporate into clinicians’ decision making. Research has shown when clinicians are engaged in the design process of smart home systems, the accuracy and efficacy of the systems are improved. We present the process undertaken by a team of nurse researchers and computer science engineers to design clinically meaningful behavior markers derived from smart home sensor data that can be used by nurses to proactively identify changes in patient status. During the first phase of design, nurse researchers qualitatively analyzed time series from smart home sensors installed in the homes of community dwelling older adults and identified patterns in these data related to significant health changes. From this analysis, we assembled a candidate list of 15 sensor-based behavior metrics, such as percent time spent in each room or frequency of bathroom use. During the second phase of design, we will build on lessons we learned from participatory design to create behavior markers and visualizations that are inspired by clinical experience. These include visualizing behavior change over time, highlighting behavioral anomalies at multiple time scales, and calculating markers that are not directly observable such as time spent out of home. Lessons learned from clinicians using the data visualizations to proactively screen for health changes in near real time will also be discussed.

